# Electrodeposition of Zinc onto Au(111) and Au(100) from the Ionic Liquid [MPPip][TFSI]

**DOI:** 10.1002/anie.202107195

**Published:** 2021-08-06

**Authors:** Fabian M. Schuett, Maren‐Kathrin Heubach, Jerome Mayer, Maximilian U. Ceblin, Ludwig A. Kibler, Timo Jacob

**Affiliations:** ^1^ Institute of Electrochemistry Ulm University Albert-Einstein-Allee 47 89081 Ulm Germany; ^2^ Helmholtz-Institute-Ulm (HIU) Electrochemical Energy Storage Helmholtzstr. 11 89081 Ulm Germany; ^3^ Karlsruhe Institute of Technology (KIT) P.O. Box 3640 76021 Karlsruhe Germany

**Keywords:** alloys, dendrites, ionic liquids, single crystals, zinc metal deposition

## Abstract

The improvement of rechargeable zinc/air batteries was a hot topic in recent years. Predominantly, the influence of water and additives on the structure of the Zn deposit and the possible dendrite formation were studied. However, the effect of the surface structure of the underlying substrate was not focused on in detail, yet. We now show the differences in electrochemical deposition of Zn onto Au(111) and Au(100) from the ionic liquid *N*‐methyl‐*N*‐propylpiperidinium bis(trifluoromethanesulfonyl)imide. The fundamental processes were initially characterized via cyclic voltammetry and in situ scanning tunnelling microscopy. Bulk deposits were then examined using Auger electron spectroscopy and scanning electron microscopy. Different structures of Zn deposits are observed during the initial stages of electrocrystallisation on both electrodes, which reveals the strong influence of the crystallographic orientation on the metal deposition of zinc on gold.

## Graphical abstract

The differences of the electrochemical deposition of Zn onto Au(111) and Au(100) from the ionic liquid *N*‐methyl‐*N*‐propylpiperidinium bis(trifluoromethanesulfonyl)imide are presented. The clearly different structures of the deposits on both gold single crystal electrodes reveal a strong influence of the crystallographic orientation on the metal deposition of zinc.

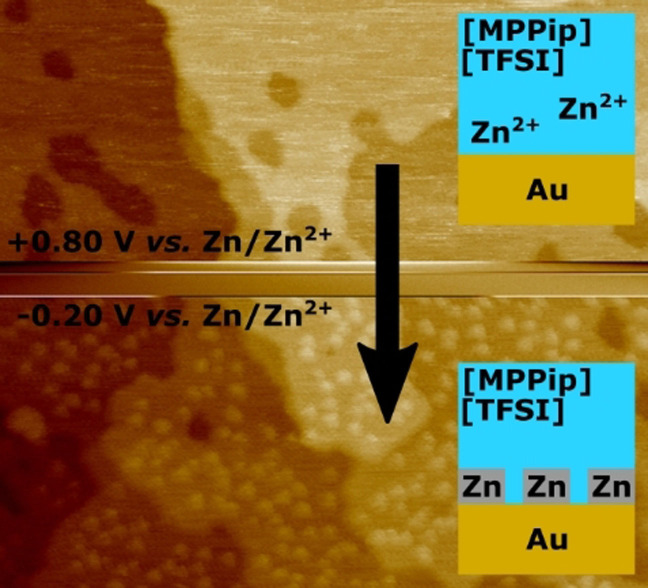

## Introduction

In recent years, the market demand for improved mobile energy devices increased tremendously. Only in 2013, five billion lithium‐ion batteries were sold to supply mobile phones, laptops, cameras, and electric vehicles, and until today this demand has grown exponentially.[^1^, ^2^, ^3^, ^4^] However, despite all research that is put into improved lithium‐based batteries, they are approximating their limit and still suffer from the initial problem of possible dendrite formation during usage.^[5]^ To satisfy the high demands, other “beyond‐lithium” battery technologies, for example on basis of sodium, magnesium, aluminum, calcium, and zinc, need to be explored.[^6^, ^7^] Especially those five alternative metals have attracted growing interest because of their cost‐effectiveness, nontoxicity, and high abundance compared to Li.[^8^, ^9^, ^10^]

Even though zinc was already applied in numerous batteries from which some are still in use today (e.g. Zn/MnO_2_ alkaline battery), most of them became outdated since 1991 by the “power source of choice”, the lithium‐ion system.[^11^, ^12^] Still, rechargeable zinc‐air batteries came recently into focus due to their distinctly higher theoretical energy density than Li‐ion batteries of up to 1086 Wh kg^−1^ (including oxygen).[^13^, ^14^, ^15^]

The development of rechargeable zinc‐air batteries pursues two main approaches: electrochemical cells on the basis of either aqueous alkaline or aprotic electrolytes.^[6]^ Though promising results of aqueous prototypes can be found,[^8^, ^16^] there are still specific problems associated with water‐based electrolytes. As the standard electrode potential of zinc is more negative than that of the hydrogen electrode, zinc electrodeposition from aqueous electrolytes is mostly accompanied by water decomposition. Therefore, even under optimum conditions, energy efficiencies of only about 60–70 % were reached.[^17^, ^18^] Additionally, the further development of these batteries has been limited due to the volatility of the electrolyte, corrosion, emergence of passivating carbonate layers (since aqueous alkaline electrolytes are very sensitive to atmospheric CO_2_) and especially dendrite formation, which is a thriving topic in Zn deposition for many years.[^17^, ^19^, ^20^, ^21^, ^22^, ^23^, ^24^, ^25^] All of these characteristics have a strongly negative impact on the electrochemical cycling stability.

To circumvent these problems, the use of ionic liquids (ILs) as aprotic electrolyte was proposed.[^10^, ^26^] This class of liquids consists of organic salts which are most commonly liquid at room temperature or at least below 100 °C.[^27^, ^28^, ^29^] They feature a high ionic and electric conductivity, low vapor pressure, non‐flammability, and comparably low toxicity.[^11^, ^29^, ^30^, ^31^] By exchange of the organic moieties, the properties of the ILs can be tuned to fit a broad variety of applications.[^27^, ^28^] Most notably is the electrochemical window of many ILs that is wide enough for the deposition of metals, which cannot be deposited from aqueous solutions.[^31^, ^32^, ^33^] One of the most investigated metals was lithium,[^5^, ^11^, ^30^] however, other metals, for example titanium^[34]^ and dysprosium,^[35]^ could be electrochemically deposited from ionic liquids as well.

Another highlight is the reversible deposition of dendrite free zinc.^[6]^ This could already be achieved by many research groups with the focus on the varying deposition behavior from different ILs with selected anion and cation combinations.[^7^, ^14^, ^18^, ^21^, ^36^, ^37^, ^38^, ^39^] In addition, the effect of additives on the zinc deposit, for example NaCl, dimethyl sulfoxide, ZnO and various Zn^2+^ salt anions was studied in detail.[^20^, ^39^, ^40^, ^41^, ^42^, ^43^] Furthermore, the influence of varying amounts of water in the IL to alter the deposition behavior, as well as the density, viscosity and conductivity was examined.[^9^, ^20^, ^38^, ^43^, ^44^, ^45^, ^46^, ^47^] The main aspect, however, is that for dendrite free zinc deposition especially the concentration of water in the electrolyte appeared to be the critical parameter.[^20^, ^21^, ^44^]

Nevertheless, despite the significant role of the electrolytic zinc bath, the initial stages and the morphology of the bulk deposits are expected to be strongly dependent on the crystallographic orientation of the underlying substrate, which has not been studied, so far. In this work, the differences between the electrochemical deposition and dissolution of zinc on Au(111) and Au(100) model electrodes from the ionic liquid *N*‐methyl‐*N*‐propylpiperidinium bis(trifluoromethanesulfonyl)imide ([MPPip][TFSI]) were investigated. Our study aims at evaluating the influence of the electrode surface structure on the initial stages of zinc deposition and on a possible dendrite formation.

We chose [MPPip][TFSI] since it was already used as electrolyte additive in Li‐ion cells to improve discharge capacities and reduce flammability as well as exothermic heat evolution. Additionally, it is stable against air and water, which would make it also suitable for use in Zn‐air cells.[^48^, ^49^, ^50^] Gold single crystals offer clean and well‐defined surfaces, which make them ideal electrodes to study ILs and to understand the relevant fundamental processes.[^31^, ^51^, ^52^, ^53^] Moreover, the above‐mentioned studies of zinc deposition on gold single crystals from different ILs focused mainly on Au(111), while ignoring the effect of the crystallographic orientation.

Basic characterization of the system was conducted using cyclic voltammetry (CV) and in situ scanning tunnelling microscopy (STM). Different structures of zinc deposits during the initial stages on both gold electrodes reveal the strong influence of the crystallographic orientation on the electrocrystallization process. In addition, many indications for an alloy formation have been detected for both single crystal surfaces. The deposition of Zn leads to a positive shift in the negative decomposition potential of [MPPip][TFSI], which limits the potential regime of Zn electrodeposition. Furthermore, Zn deposits fabricated by several potentiodynamic cycles were examined by Auger electron spectroscopy (AES) and scanning electron microscopy (SEM). There were no signs of dendrite formation during Zn deposition on both Au single crystal electrodes under the experimental conditions.

## Results and Discussion

Prior to zinc deposition, the electrochemical stability window of the pure ionic liquid was determined to 4.20 V on both gold electrodes. However, the decomposition potentials have an offset of 0.15 V to each other. The stability window of Au(111) in [MPPip][TFSI] has been determined to range from −1.55 V to +2.65 V vs. Zn/Zn^2+^. These potentials are shifted negatively to −1.70 V and +2.50 V vs. Zn/Zn^2+^ for Au(100) (see Figure S1 in the Supporting Information (SI)). [MPPip][TFSI] on gold electrodes shows cathodic decomposition below the stability region, while anodic gold oxidation/dissolution takes place at positive potentials.

**Figure 1:**
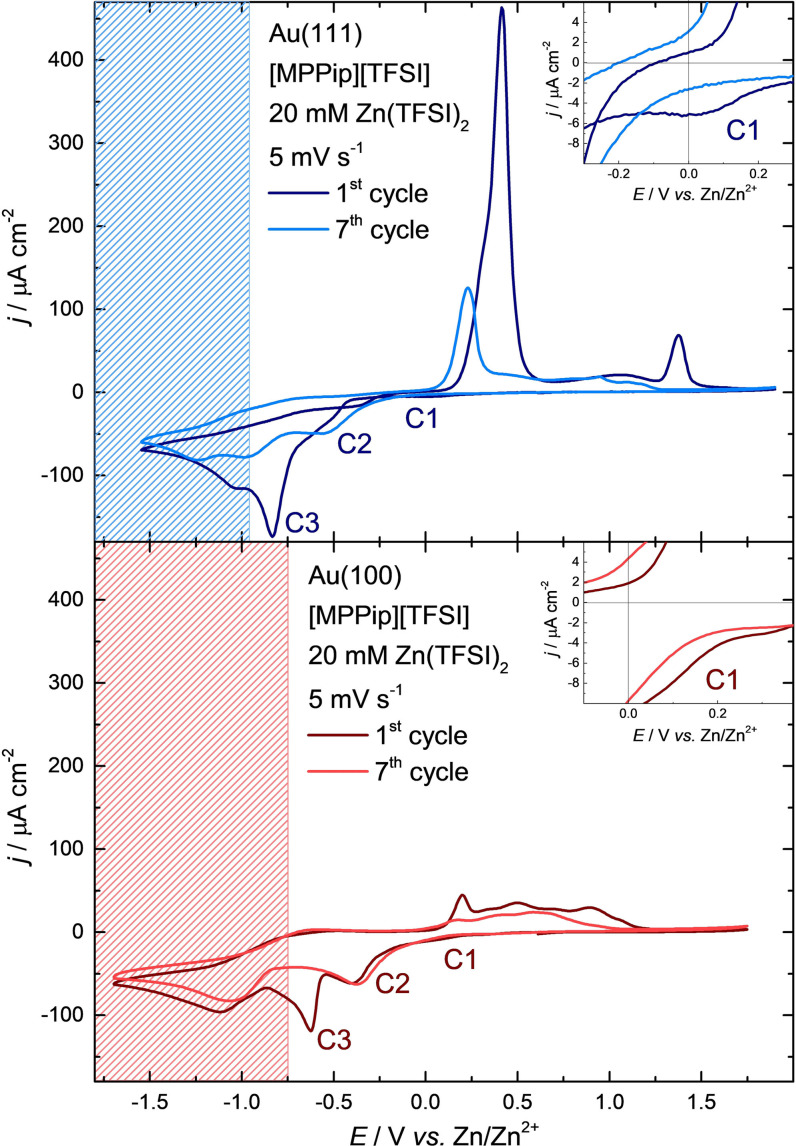
Current‐density–potential curves for Au(111) and Au(100) in contact with [MPPip][TFSI] + 20 mM Zn(TFSI)_2_ between −1.55 V and +1.90 V (top, blue) and between −1.70 V and +1.75 V (bottom, red) vs. Zn/Zn^2+^, respectively. Scan rate: 5 mV s^−1^. The graphs show the 1^st^ and 7^th^ voltammetric cycle. The inset displays a magnification of the UPD region. The shaded areas mark the decomposition region of [MPPip][TFSI] on the deposited zinc layers.

Based on these results, identical negative potential limits were chosen for the electrochemical analysis of the Au electrodes in [MPPip][TFSI] + 20 mM Zn(TFSI)_2_. The positive potential limit, however, was decreased to +1.90 V vs. Zn/Zn^2+^ for Au(111) and to +1.75 V vs. Zn/Zn^2+^ for Au(100) to avoid undesired processes, such as surface oxidation, dissolution of the gold surface, and decomposition of the electrolyte at positive potentials. Current density‐potential curves of Au(111) and Au(100) in contact with [MPPip][TFSI] + 20 mM Zn(TFSI)_2_ are shown in Figure 1.

In the first cycle, two main deposition peaks can be distinguished for both gold electrodes (C2 and C3). In addition, both electrodes show a small cathodic peak (C1), starting at approximately 0.20 V positive of the equilibrium potential. While C1 appears as a distinct peak for Au(111), this process merges directly into the first main deposition peak (C2) for Au(100). Therefore, for the charge determination of C1 on Au(100) the peak was integrated from the beginning of C1 until 0 V vs. Zn/Zn^2+^. C1 can be either caused by underpotential deposition (UPD) or a non‐zinc‐related process (compare the peaks between 0.0 V and 0.5 V of the pure IL in Figure S1 in the SI). Under the assumption of an UPD the Zn coverage for C1 was estimated to be 0.41 monolayers (ML) on both gold surfaces. ML values are calculated with the charge density, which can be obtained by integration of the current density over the measurement time (182.5 μC cm^−2^ and 159.3 μC cm^−2^ for C1 on Au(111) and Au(100), respectively), divided by 2 (required electrons per deposited zinc atom) and the particular monolayer equivalent of each gold surface (222 μC cm^−2^ for Au(111) and 192 μC cm^−2^ for Au(100)). This is in a reasonable order of magnitude for an UPD and in agreement with Zn UPDs that have already be reported for the zinc deposition on gold from aqueous and ionic electrolytes.[^21^, ^36^] However, we were only able to observe a single UPD peak instead of multiple ones, as reported for the zinc deposition from other ionic liquids.[^18^, ^36^] Despite the distinct C1 peak in the CV on Au(111), the current density does not completely go back to zero after 0 V vs. Zn/Zn^2+^. Instead, it stays at approximately 5–8 μA cm^−2^ and continues as a slight hump of C2 until at −0.38 V vs. Zn/Zn^2+^ suddenly the C2‐peak starts. A possible reason that C1 merges directly into C2 on Au(100) but forms a rather separate peak on Au(111) could be, that Au(100) has a more open surface structure in the face centered cubic lattice and should therefore provide more stable crystallization sites for Zn atoms. Another identification for this is the hysteresis for the zinc deposition that can only be observed on Au(111). A similar behavior was already observed for other ionic liquids, which indicates a considerable overpotential for further zinc deposition after the Zn UPD.[^20^, ^39^, ^46^]

Further analysis of the system reveals that the electrodeposition of zinc results in a positive shift of the negative decomposition potential of [MPPip][TFSI]. These potentials were determined to be −0.95 V and −0.75 V vs. Zn/Zn^2+^ for Au(111) and Au(100), respectively. The areas negative of these potentials are shaded blue and red in Figure 1. It needs to be considered, that when scanning towards negative potentials through C1‐C3, approximately 20 ML of Zn were deposited on both gold surfaces (ca. 8.85 mC cm^−2^ on Au(111) and ca. 8.60 mC cm^−2^ on Au(100)). Thus, at potentials negative of C3 the Au surfaces are considerably plated with Zn and behave similar to a Zn bulk electrode. For this reason, the positive shift of the decomposition potential can be explained by the decomposition of the IL on the deposited Zn films at less negative potentials than on the Au surfaces. Two main reasons can be given for this behavior. First, the previous negative stability potential of the electrolyte on gold is shifted towards more negative potentials after passing through the decomposition area: In Figure S1 in the SI the cathodic current density slowly begins to rise at the negative end of the electrochemical window, due to the beginning decomposition of [MPPip][TFSI] on Au. However, this is not the case in Figure 1, where the scan direction changes after reaching the negative reversal potential, without any hint of rising cathodic currents. This can be explained by decomposition products of the IL that formed on the Zn‐covered surface at more positive potentials. These decomposition products are now insulating the electrode surface from the electrolyte, thus preventing further reduction. A similar effect was previously observed by Tułodziecki et al. In their study, a lithium catalyzed breakdown of TFSI containing ILs was examined after scanning to negative potentials.^[54]^ In contrast to the here formed SEI (solid electrolyte interface), which is beneficial in lithium and sodium‐based batteries and still allows diffusion of metal ions to the electrode,[^55^, ^56^, ^57^, ^58^, ^59^] the decomposition layer of [MPPip][TFSI] seems to be impermeable for zinc ions. Second, this surface insulation leads to a continuous decrease of the anodic and cathodic current densities with further cycling. This can also be seen in the development of the current density potential curves in Figure 1 from the 1^st^ to the 7^th^ cycle.

Measurements of the pure IL on a polished poly zinc plate confirmed a more positive decomposition potential of [MPPip][TFSI] on Zn compared to Au. The decomposition potential of [MPPip][TFSI] with beginning insulation of the zinc electrode could be determined to be about −0.70 V vs. Zn/Zn^2+^ (see Figure S2 in the SI). This decomposition potential is even a bit more positive compared to the decomposition potential of Zn deposited onto Au. The reason for this could be the high number of kinks and steps of the freshly polished Zn plate, compared to the rather flat deposited structures on the gold single crystals. Alternatively, the deposited zinc structures might be stabilized by the gold surface, for example, by the formation of an alloy, thus being less reactive to the ionic liquid.

An insulation layer on top of the in the first cycle deposited zinc also affects the stripping in the subsequent positive sweep. The comparison of the cathodic and anodic charge (calculated without cathodic decomposition currents) results in 2:1 anodic versus cathodic on Au(111). This is another evidence for reduced organic decomposition products on the electrode surface, which are getting oxidized in the positive scan. On Au(100), however, the reversibility could be determined to ca. 75 %. The reason for this might be that the decomposition of [MPPip][TFSI] starts here at more positive potentials as on Au(111) and at the same time the potential was swept 150 mV more negative, which could have led to a thicker and more insulating layer.

**Figure 2:**
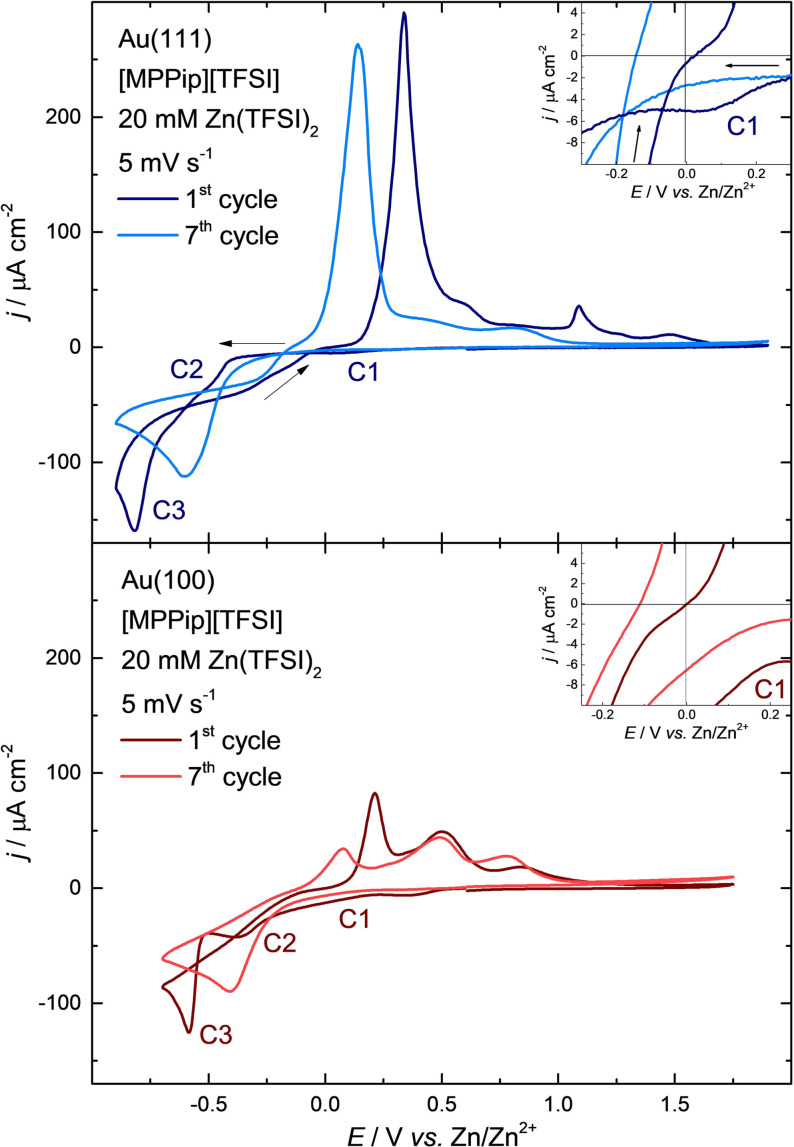
Current density‐potential curves for Au(111) and Au(100) in contact with [MPPip][TFSI] + 20 mM Zn(TFSI)_2_ between −0.90 V and +1.90 V (top, blue) and between −0.70 V and +1.75 V (bottom, red) vs. Zn/Zn^2+^, respectively. Scan rate: 5 mV s^−1^. The graphs show the 1^st^ and 7^th^ voltammetric cycle. The inset displays a magnification of the UPD region. The arrows in the top image indicate the scan direction for the Au(111) voltammogram.

Increasing the negative potential limit to −0.90 V vs. Zn/Zn^2+^ for Au(111) and to −0.70 V vs. Zn/Zn^2+^ for Au(100) enables the observation of zinc deposition and stripping without decomposition of the electrolyte (see Figure 2). While the beginning of the negative sweep in Figure 2 still looks the same as for the large window in Figure 1, a few obvious differences can directly be detected: On Au(111), the hysteresis of the depositing zinc negative of the equilibrium potential is not anymore limited to the first cycle. Despite the missing UPD from the second cycle onwards, the overpotential only decreases very slowly within the further cycles. The amount of deposited zinc could now be estimated to approximately 34 ML (C1=184.4 μC cm^−2^, C2=1788.4 μC cm^−2^, C3=13 280.6 μC cm^−2^) on Au(111) and to approximately 27 ML (C1=158.0 μC cm^−2^, C2=2355.6 μC cm^−2^, C3=7861.6 μC cm^−2^) on Au(100), since decomposition products do not anymore slow down the electrocrystallization process. In addition, besides two minor distinctions, the further cycles appear very similar compared to the first cycle with the smaller potential window. The first difference is that within the first few cycles the three distinct deposition peaks change to only one deposition peak on both gold electrodes, which is a sign of a decreasing amount of uncovered/unchanged gold surface in comparison to the beginning of the experiment. Second, the beginning of the anodic peak shifts slowly to slightly more negative potentials; within the first seven cycles by −0.10 to −0.13 V vs. Zn/Zn^2+^.

Comparison of the anodic and cathodic charge for the small potential window on Au(111) resulted in slightly more than 80 % reversibility (anodic/cathodic=12.8 mC cm^−2^/15.3 mC cm^−2^=0.84 for the first cycle and 10.8 μC cm^−2^/13.2 μC cm^−2^=0.82 for later cycles). For Au(100) the reversibility could be determined to just below 60 % in the first cycle (5.9 mC cm^−2^/10.4 mC cm^−2^=0.57) and to roughly above 70 % (5.2 mC cm^−2^/7.3 mC cm^−2^=0.71) in the following cycles. With the assumption that no superimposed signals from decomposition products of the electrolyte are present, and because it is known that gold and zinc are mixing well,[^18^, ^21^, ^36^, ^46^, ^60^, ^61^] we propose the formation of a Zn‐Au‐alloy as a possible reason for the charge imbalance. This is supported by the previously mentioned change of the three deposition peaks to one peak with continuous cycling and thus, the decreasing amount of uncovered gold surface. Furthermore, different alloys with varying surface energies can form on both surfaces which would explain the divergent reversibility on both surfaces. Three main alloys between zinc and gold are known: the hexagonal Au_1.2_Zn_8.8_ phase as well as the cubic AuZn and AuZn_3_ phases. In situ X‐ray diffraction (XRD) revealed that on polycrystalline gold films all three alloys are formed, on an Au(111) surface, however, only the formation of the hexagonal Au_1.2_Zn_8.8_ alloy could be observed.^[18]^

**Figure 3:**
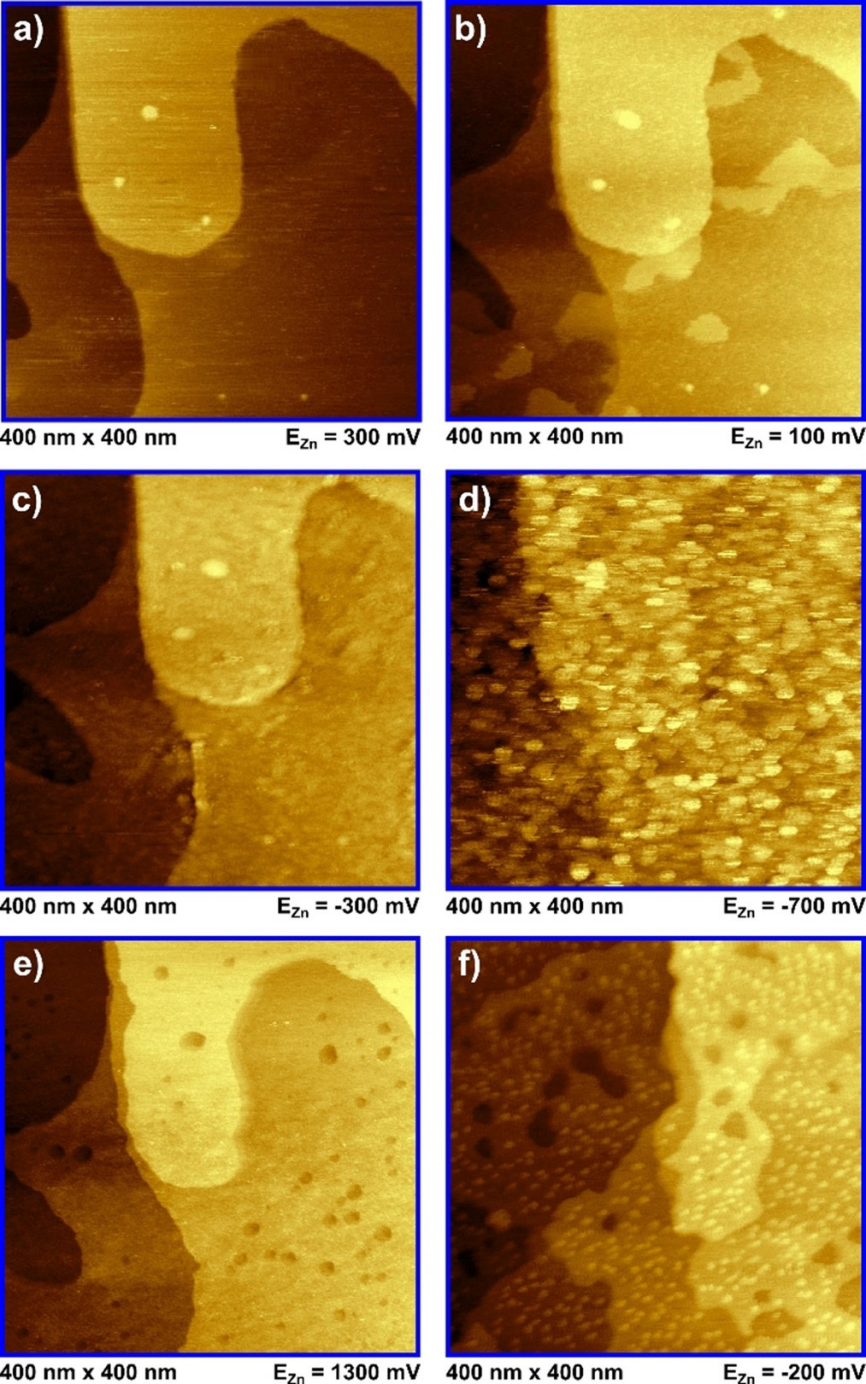
In situ STM images for Au(111) in [MPPip][TFSI] + 20 mM Zn(TFSI)_2_. All scans were measured from bottom to top. a) pristine gold surface before the first deposition. b) UPD region with Zn islands. c) completion of Zn adlayer. d) Zn bulk deposition. e) sweep to positive potentials: formation of 1–2 monolayer deep holes after all zinc on top of the gold surface was stripped. f) beginning of the deposition in the 2nd cycle after holding at positive potentials for approximately 15 minutes.

Figure 3 shows in situ STM images of zinc deposition and dissolution on Au(111) at different potentials. Figure 3 a displays the pristine and thermally reconstructed Au(111) surface before the deposition process. This area was chosen because it shows wide gold terraces with a few monoatomic high steps. Figure 3 b was recorded in the UPD regime. Here the formation of flat islands on the terraces can be observed until a coverage of roughly half a monolayer is obtained. This corresponds to the calculated current densities for C1, which is another indication of zinc UPD. The height of these islands was determined to be 0.14 nm (see Figure S3 a) in the SI for height profiles of Figure 3 b), which is much lower than the monoatomic step height of Au(111) with 0.24 nm.[^21^, ^62^] The height of a monoatomic high zinc adlayer on Au(111) was determined to 0.22 nm, when studying the zinc deposition from an AlCl_3_ containing IL.^[36]^ However, in this case, three distinct UPD peaks were observed instead of only one and it was already shown that aluminum and zinc can be co‐deposited from ionic liquids.^[40]^ Furthermore, similar to our study, zinc UPD on Au(111) from 1‐ethyl‐3‐methylimidazolium trifluoromethylsulfonate ([EMIm][TfO]) appears to have a smaller height than the gold monoatomic high steps as well. Even though Liu et al. do not state an explicit value for the height of deposited zinc layers in their study.^[21]^ At the beginning of C2, the observed UPD islands slowly start merging by further Zn deposition, followed by additional uniform layer growth on top (Figure 3 c). Despite this growth of further Zn layers, the surface structure of the underlying gold surface can still be recognized. During imaging, it was not possible to determine the number of deposited layers in this step. However, evaluation of the CVs resulted in the deposition of approximately 3–4 ML. Scanning into the bulk deposition regime C3 resulted in the growth of many small, equally distributed and overlapping islands (Figure 3 d). Stepping to positive potentials, the stripping of the deposited zinc could be observed. Despite some blurriness, the examined surface appeared similar to the pristine gold surface observed in the beginning of the experiment, shown in Figure 3 a. However, holding the potential at 1.30 V vs. Zn/Zn^2+^ for some minutes showed the slow formation of one to two monolayer deep holes on the suspected gold surface together with etching patterns at the step edges (Figure 3 e). This is another indication for already proposed formation of a gold/zinc alloy and thus the reason for the imbalance in cathodic and anodic charge, which was observed in the CVs (see Figure S3 b) in the SI for height profiles of Figure 3 e). Furthermore, very similar etching structures have been observed for the dissolution of Au‐Zn‐alloys from other ILs.[^21^, ^36^] A potential step back to −0.20 V vs. Zn/Zn^2+^ indicates the differences between the first and following cycles. Figure 3 f shows the immediate formation of equally distributed small zinc islands instead of a UPD and layer‐like growth, as it was observed in the first cycle.

**Figure 4:**
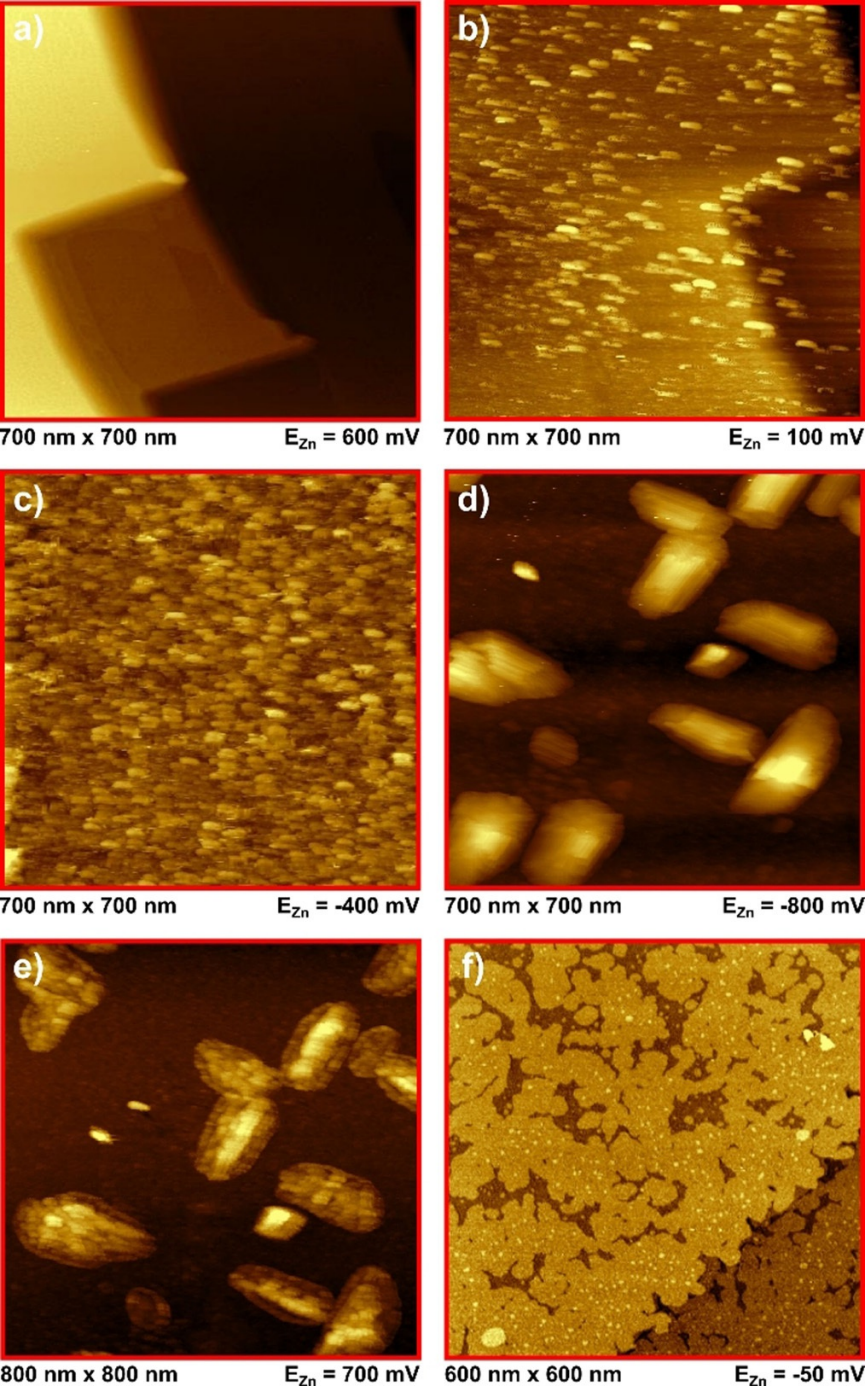
In situ STM images for Au(100) in [MPPip][TFSI] + 20 mM Zn(TFSI)_2_. All scans were measured from bottom to top. a) pristine gold surface before the first deposition. b) UPD region with first Zn islands. c) completion of Zn adlayer. d) Zn bulk deposition. e) sweep to positive potentials: remnants of the clusters stay on the surface. f) beginning of the deposition in the 2nd cycle after holding at positive potentials for approximately 25 minutes.

In situ STM images for the zinc deposition and dissolution on Au(100) are shown in Figure 4. Similar as before for Au(111), Figure 4 a displays the pristine and thermally reconstructed Au(100) surface before the beginning of the first deposition process. In the UPD regime (Figure 4 b) the first difference between the deposition on both surfaces can be detected. Here, the formation of many small monolayer‐high islands can be observed, instead of a few broader ones as on Au(111) (see Figure S4 a) in the SI for height profiles of Figure 4 b). Similar to the results for Au(111) no multilayer growth has been observed, which indicates the formation of a single, albeit incomplete, monolayer. This island‐like deposition process continued very slowly until most of the surface was covered by those islands. In accordance with the missing separation between the UPD‐peak C1 and C2 in the CVs of Au(100) (Figure 1 and Figure 2), a smooth transition to a completely island‐covered surface could be observed in the STM by lowering the potential (Figure 4 c). A varying island structure in every further frame implies a subsequent deposition of more zinc islands on top of previous ones in the C2 regime on Au(100); similar to C3 on Au(111). This again fits the current‐potential curves, which resulted in deposition of approximately 6–8 ML. At C3 the deposition changes towards an intense cluster growth with a layered morphology. Holding the potential at −0.80 V vs. Zn/Zn^2+^ for slightly more than one hour led to steady growth of clusters (Figure 4 d) until a height of up to 8 nm (see Figure S4 b) in the SI for height profiles of Figure 4 d). It can be observed that these clusters exhibit two orthogonally preferential growth directions, which agrees with the rectangular symmetry of the underlying Au(100) surface. However, even before the start of C3, the gold surface is covered by at least 6 ML of zinc that would expect to mask the underlying gold structure. The formation of one of the two cubic zinc‐gold alloy phases (AuZn or AuZn_3_)^[18]^ between the deposited Zn islands and the Au(100) surface could be a possible explanation. These cubic alloys could then act as nucleation seeds for the clusters, causing the orthogonal symmetry. During and after the stripping process remnants of those clusters stay on the surface (Figure 4 e). Even at potentials positive of the anodic peaks in the CVs, those remnants stay on the surface for nearly half an hour. This is an additional indication for the supposed alloy formation that causes the irreversibility between the cathodic and anodic sweeps in the current‐potential curves. Holding the potential just positive of the anodic peaks for some time reveals similar pitting patterns as observed on the Au(111) electrode and shown in Figure 3 e. Figure 4 f shows an image after stepping back to −0.05 V vs. Zn/Zn^2+^. At a first glance, the Au(100) surface looks quite similar to the Au(111) surface at the beginning of the second cycle. However, when examining the images in more detail, it can be recognized that the Zn islands on Au(100) are smaller than the ones on Au(111) and additionally square in shape. Furthermore, a few larger islands can be detected on Au(100) as well (bright spots in Figure 4 f), which appear to mark the beginning of the zinc cluster growth.

**Figure 5:**
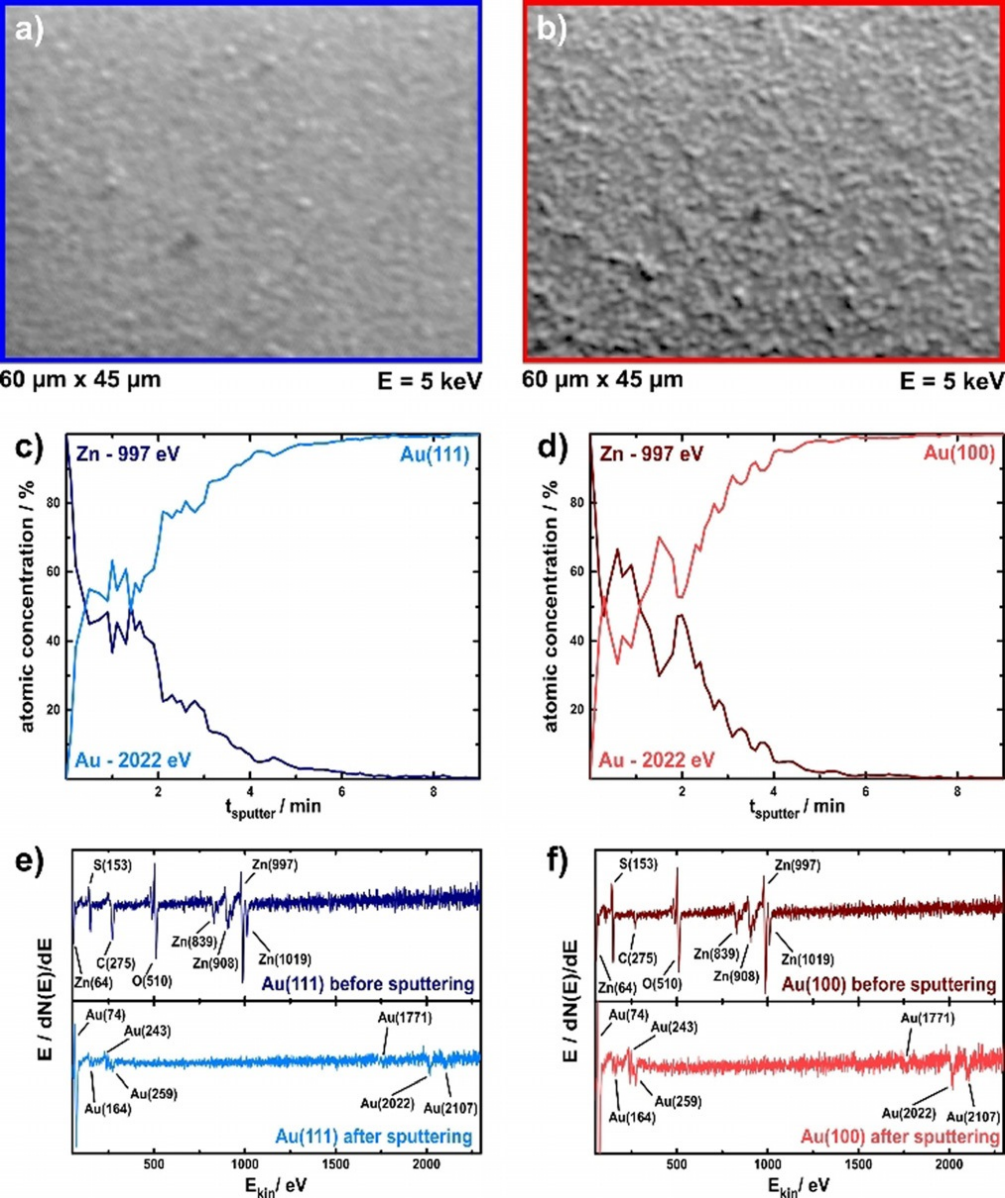
SEM and AES investigation after ca. 2 hours of cycling in the zinc deposition range with incomplete Zn stripping. a) and b) SEM images of the Zn deposit on Au(111) and Au(100), respectively. Depth profile sputter experiments of the deposited zinc on the Au(111) (c)) and Au(100) (d)) surface. e) and f) AES spectra of the surfaces before and after sputtering. All peaks have been assigned with published data.^[63]^

Despite the differences in the deposition and stripping of zinc on both gold surfaces, no evidence of dendrite formation could be found for either. This is in contrast to the initial stages of dendrite formation that could be observed for the lithium deposition on Au(111) from the very same IL due to incomplete Li dissolution after the formation of a surface alloy.^[5]^ Therefore, further cycling experiments with incomplete zinc stripping within the stability range of the IL were conducted to check for signs of dendrite formation on basis of the electrode surface structure. In addition, such cycling experiments are of interest for subsequent possible battery applications. Both electrodes were cycled at 5 mV s^−1^ between −0.70 V vs. Zn/Zn^2+^ and 0.45 V vs. Zn/Zn^2+^ for approximately two hours. After these experiments, a shiny metallic zinc coating visible to the naked eye was obtained on both gold single crystals. SEM images of those bulk deposits again show the observed differences in the deposition. The zinc surface on Au(111) (Figure 5 a) appears flatter and more homogeneous compared to the zinc surface on Au(100) (Figure 5 b). In addition, an island‐like structure similar to the one observed in the STM (Figure 3 d) can still be surmised. The deposited Zn surface on Au(100) appears rougher, which is most likely be caused by the previously observed cluster growth. Despite the different structures of the zinc plating and the differences in the deposition behavior, no signs of dendrite formation could be found on either electrode. Instead, a solid and equal plating was achieved on both surfaces. This is different from other non‐aqueous liquids as acetonitrile or propylene carbonate from which only sponge‐like “nanowires” could be deposited.^[7]^

AES depth profile sputter experiments of those samples from the cycling experiment reveal similar coating thicknesses on both surfaces (Figure 5 c and d). The surfaces have been continuously sputtered with argon ions for 6 s between every AES measurement. The shown curves in Figure 5 c and d are plots of the relative intensity of the Zn peak at 997 eV and the Au peak at 2022 eV over the complete sputter time. Both sputter profiles show a plateau with similar amounts of zinc and gold after the first few zinc layers have been removed; between roughly 0.6 min and 1.8 min of sputter time. Constant peak intensities indicate a homogeneous distribution of gold and zinc atoms in this sputter depth, which is another indication of the formation of Zn/Au alloys. After roughly 1.8 min of sputtering the intensity of both peaks is changing again, until finally the Zn peak has vanished, and the Au peak is the only remaining one. In general, it was found that the alloy thickness on Au(100) is slightly thicker than that on Au(111). The displayed representative depth profiles show the constant plateau until 1.6 min for Au(111) and 2 min for Au(100). The thicker alloy depth seems to be the reason for the poorer reversibility of the zinc deposition and stripping processes on Au(100) compared to the ones on Au(111). Figure 5 e and f show AES measurements of the sputtering area before and after the sputter processes. Before sputtering, the experiment shows no gold peaks, but intense Zn peaks and some IL residues from the prior deposition. After sputtering, only signals from the gold electrode remain. Assignment of the Auger peaks was conducted according to literature.^[63]^

## Conclusion

While the voltammetric peaks for the initial stages of zinc deposition onto Au(111) and Au(100) from [MPPip][TFSI] are very similar, the results obtained by in situ scanning tunnelling microscopy clearly show different growth processes and morphologies of the corresponding Zn deposits. In the UPD regime the formation of large and flat island areas can be observed on Au(111), in contrast to many smaller islands on Au(100). The Zn bulk deposition on Au(111) appears as a homogeneous island‐covered surface, whereas the formation of big Zn clusters can be observed on Au(100). This reveals the strong influence of the crystallographic orientation for Zn electrodeposition on different Au single crystal electrode surfaces. Zn plating leads to a positive shift in the negative decomposition potential of the IL, which limits the potential window for Zn electrocrystallization. In contrast to the usual exponential progression of the current density for decomposition processes, the degradation of [MPPip][TFSI] is characterized by a separate voltammetric peak and self‐limiting due to the formation of a zinc impermeable SEI. While in situ STM as well as AES sputter experiments indicate alloy formation during the zinc deposition on Au, a complete and solid plating was found upon continued potential cycling. In situ STM results also showed that complete stripping of the formed zinc and gold alloys is kinetically hindered at positive potentials, which explains the poor reversibility of both systems while cycling at 5 mV s^−1^. Furthermore, no signs of dendrite formation during zinc deposition were detected for both Au single crystal electrodes. This leads to the conclusion, that despite the influence of the crystallographic orientation of the Au single crystals on the Zn adlayer structure, dendrite formation is absent if the amount of water present in the electrolyte is kept to a minimum.

## Conflict of interest

The authors declare no conflict of interest.

## Supporting information

As a service to our authors and readers, this journal provides supporting information supplied by the authors. Such materials are peer reviewed and may be re‐organized for online delivery, but are not copy‐edited or typeset. Technical support issues arising from supporting information (other than missing files) should be addressed to the authors.

Supporting Information
