# Supplementary material for: Electrodeposition of Zinc onto Au(111) and Au(100) from the Ionic Liquid [MPPip][TFSI]
Source: Angew Chem Int Ed Engl. 2021 Aug 6;60(37):20461–8. doi: 10.1002/anie.202107195 (PMC8456931; doi:10.1002/anie.202107195)
Supplement: Supplementary file 1 — Supporting Information [file ANIE-60-20461-s001.pdf]

Angewandte Chemie
Eine Zeitschrift der Gesellschaft Deutscher Chemiker

Supporting Information

Electrodeposition of Zinc onto Au(111) and Au(100) from the Ionic Liquid [MPPip][TFSI]

*Fabian M. Schuett, Maren-Kathrin Heubach, Jerome Mayer, Maximilian U. Ceblin, Ludwig A. Kibler, and Timo Jacob**

anie_202107195_sm_miscellaneous_information.pdf

SUPPORTING INFORMATION

Table of Contents

Additional Experimental Results……………………………………………………………………………2

Figure S1 ……………………………………………………………………………………………2

Figure S2 ……………………………………………………………………………………………3

Figure S3 ……………………………………………………………………………………………4

Figure S4 ……………………………………………………………………………………………5

Experimental Procedures……………………………………………………………………………6

Preparation of the Electrolyte…………………………………………………………………6

Preparation of the Electrodes…………………………………………………………………6

Preparation of STM Tips………………………………………………………………………6

CV and STM Measurements…………………………………………………………………6

Auger Measurements and SEM Imaging……………………………………………………6

Author Contributions…………………………………………………………………………………7

Additional Experimental Results

Current Density-Potential Curves of the Gold Surfaces in Pure and Zinc Free [MPPip][TFSI]

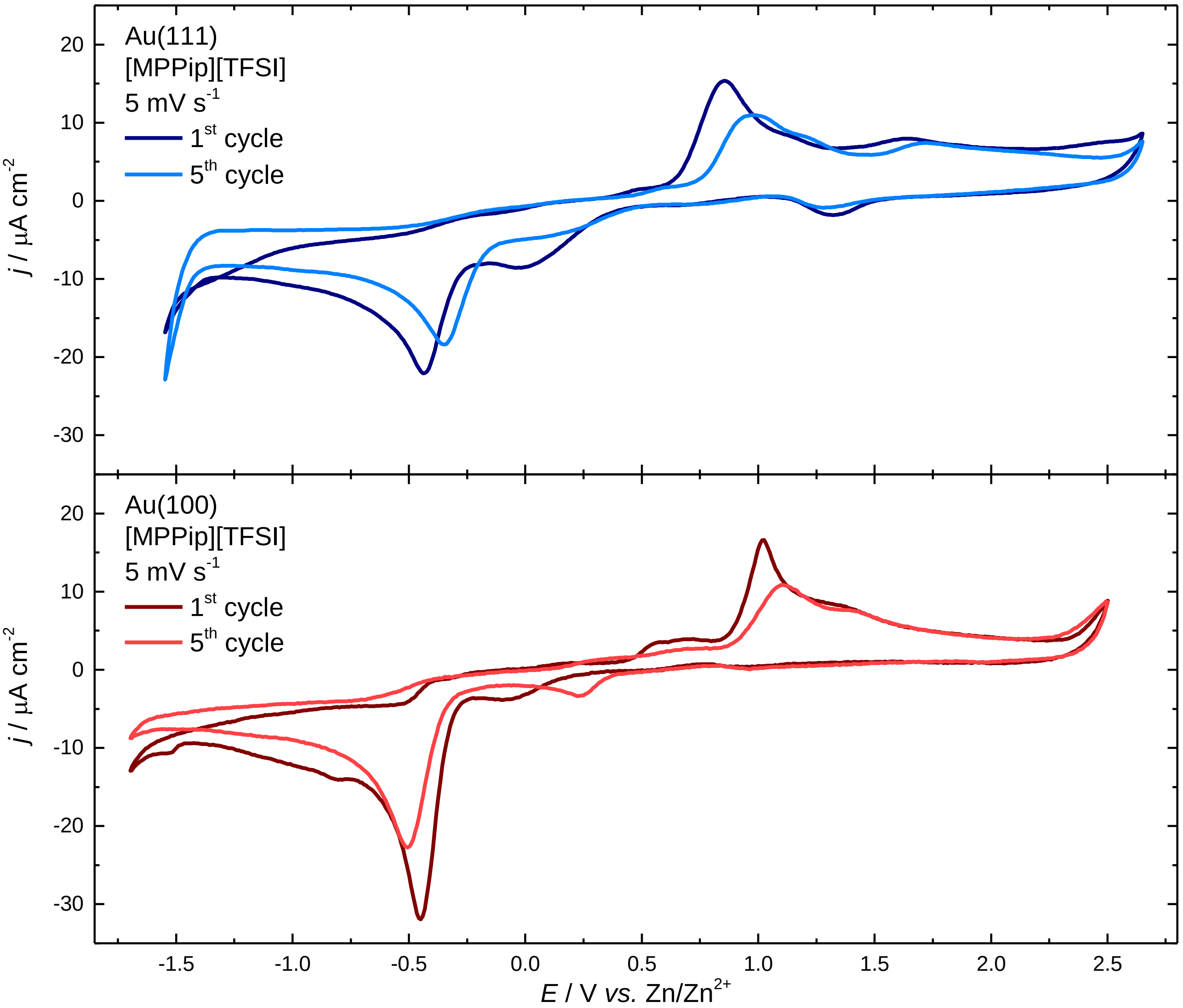

Figure S1. Current density-potential curves for Au(111) and Au(100) in contact with [MPPip][TFSI] between -1.55 V and +2.65 V (top, blue) and between -1.70 V and +2.50 V (bottom, red) *vs.* Zn/Zn^{2+}, respectively. Scan rate: 5 mV s^{-1}. The graphs show the 1st and 5th voltammetric cycle. The main cathodic and main anodic peak at ~-0.5 V and ~+0.9 V *vs.* Zn/Zn^{2+}, respectively, can in both CVs be attributed to the reaction of residual water in the IL that could not be removed by the drying process.

Current Density-Potential Curves of a Polished Zinc Plate in Pure [MPPip][TFSI]

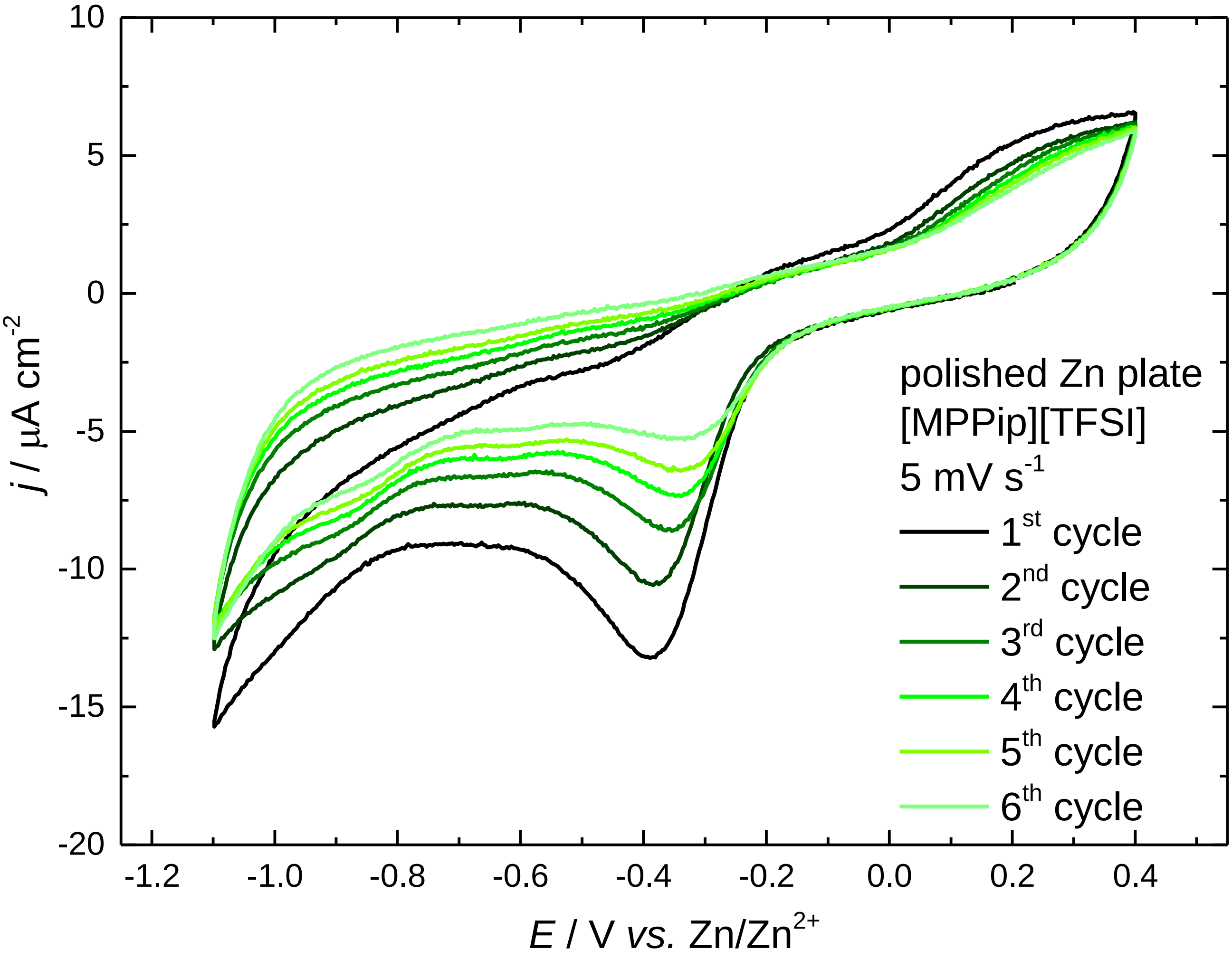

Figure S2. Current density-potential curves for a freshly polished Zn plate in contact with [MPPip][TFSI] between -1.1 V and +0.4 V *vs.* Zn/Zn^{2+}. Scan rate: 5 mV s^{-1}. The graph shows the 1st to the 6th voltammetric cycle. The first main cathodic peak at ~-0.4 V *vs.* Zn/Zn^{2+} can be attributed to the reaction of residual water in the IL that could not be removed by the drying process. The second cathodic peak represents the decomposition of [MPPip][TFSI] on the zinc plate, which is causing the insulation of the electrode. The beginning of this peak can be determined as the maximum between both cathodic peaks at approximately -0.7 V *vs.* Zn/Zn^{2+}.

Height Profiles of Figure 3

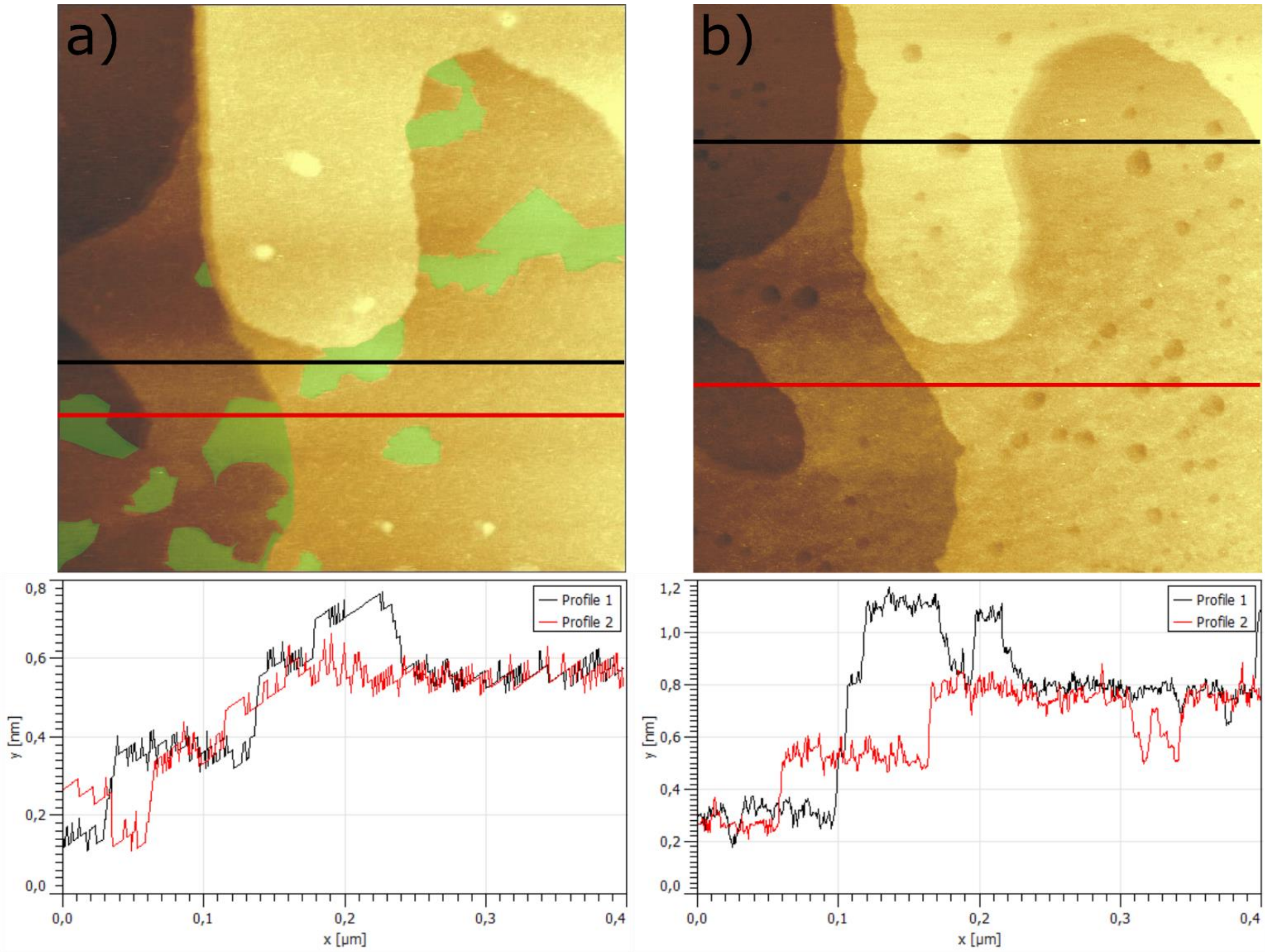

Figure S3. Height Profiles of STM images, shown in Figure 3 in the main paper. a) Height profiles of Figure 3 b). The zinc UPD islands are highlighted in green for an easier correlation to the profiles. b) Height profiles of Figure 3 e).

SUPPORTING INFORMATION

Height Profiles of Figure 4

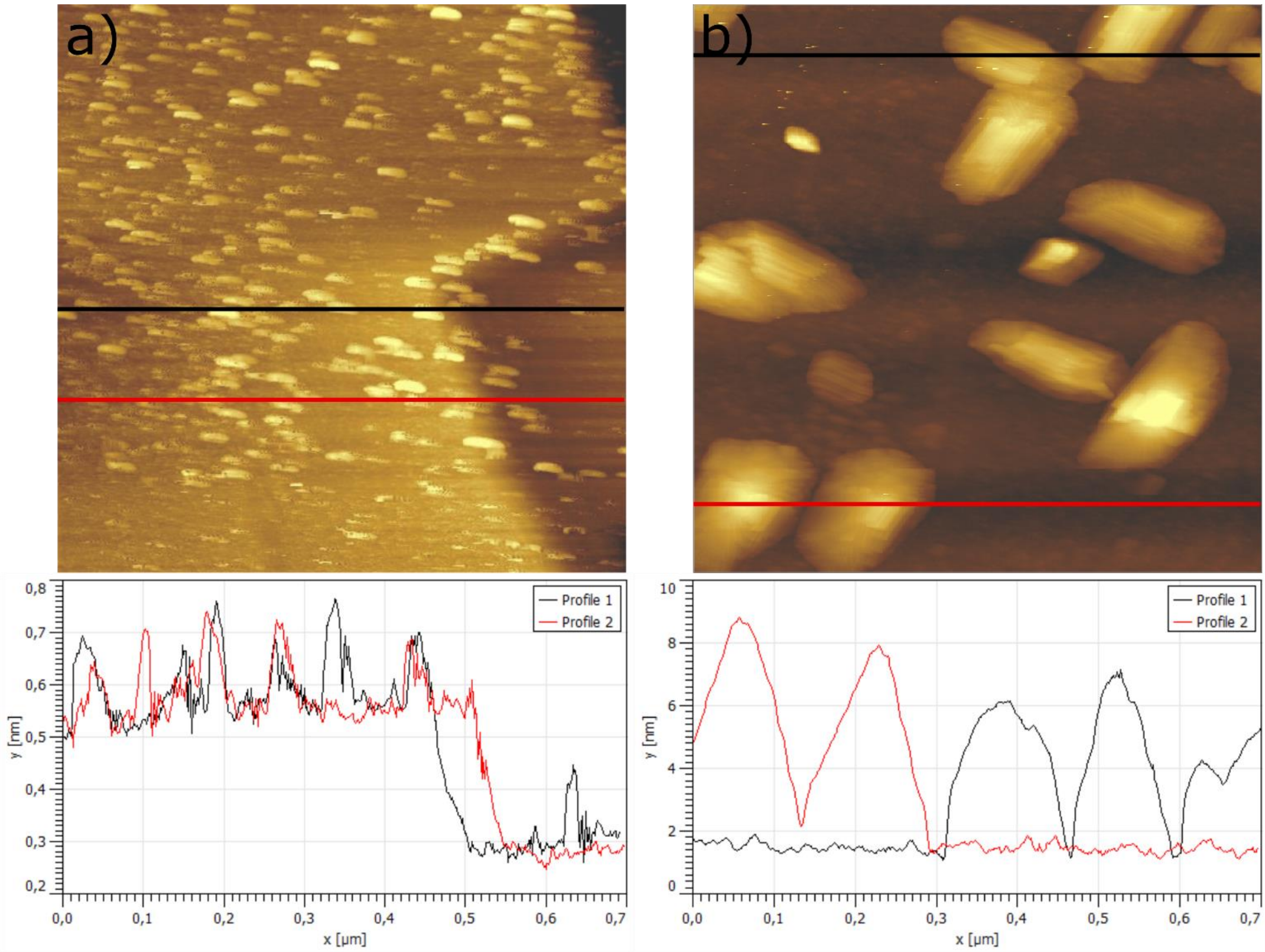

Figure S4. Height Profiles of STM images, shown in Figure 4 in the main paper. a) Height profiles of Figure 4 b). b) Height profiles of Figure 4 d).

Experimental Procedures

Preparation of the Electrolyte

Before usage the IL [MPPip][TFSI] (>99%, IoLiTec) was dried in vacuum for 24 hours at elevated temperatures (80 °C). For the zinc electrolyte 0.02 $molL^{-1}$ $Zn(TFSI)_2$ (>99.5%, Solvionic) was added to the pre-dried IL and again heated at 80 °C for 24 h. After preparation the electrolytes were stored in a MBRAUN LABstar glove box in nitrogen atmosphere containing less than 0.5 ppm water and oxygen.

Preparation of the Electrodes

The Au(111) and Au(100) single crystals (both 12 mm diameter and 2 mm thickness, MaTecK GmbH) where used as working electrodes for all measurements. Before each measurement both crystals where annealed in a furnace (Carbolite CWF 1200) at 960 °C for 2 hours to obtain a thermally reconstructed surface. After each measurement the crystals were firstly rinsed with acetone and ultra-high purity (UHP) water (water purification system - Sartorius Arium 611 UV; TOC ≤ 3 ppb) and secondly submerged in concentrated nitric acid for about 1-2 hours to remove all zinc and IL remnants. Afterwards the crystals were again rinsed thoroughly with UHP water.

Platinum wires (MaTecK GmbH) have been used as counter (CE) and quasi reference electrode (RE) for the *in-situ* STM measurements. The CE and RE were flame-annealed in a hydrogen flame to red heat and subsequently cooled in a nitrogen stream before each measurement. After each measurement the electrodes were rinsed with acetone and UHP water.

For the voltammetric measurements, a Zn sheet (99.99+%, MaTecK GmbH) was used as RE which was polished in a MBRAUN LABstar glove box in nitrogen atmosphere containing less than 0.5 ppm water and oxygen, prior to each measurement to provide an oxide-free surface. Zinc working electrodes were prepared the same way.

Preparation of STM Tips

STM tips have been prepared in house from Pt/Ir wires (80:20, MaTecK GmbH) through etching in 3.5 M NaCN and coating with BASF electrophoretic paint (ZQ84 3225) to minimize Faraday currents.

CV and STM Measurements

CV measurements were performed in a MBRAUN LABstar glove box in nitrogen atmosphere containing less than 0.5 ppm water and oxygen. The CVs have been recorded with a Zahner IM6 potentiostat controlled by the corresponding Thales Z 1.20 USB software. CV and STM measurements have been carried out in in-house designed cells for volumes of 200 µL made from KelF and Teflon. *In-situ* STM measurements have been performed with a Veeco Multimode8 Scanning Probe Microscope controlled by a Nanoscope 5 controller. For the electrochemical imaging, a Bruker Universal Bipotentiostat was used. All images have been recorded in nitrogen atmosphere and with tip currents between 1-7 nA in constant current mode.

Auger Measurements and SEM Imaging

AES measurements and SEM imaging have been performed with a PHI 660 Scanning Auger Nanoprobe from Perkin Elmer Corp., Physical Electronics Division. As electron gun and Auger electron analyser, a PHI 25-120 Electron Gun and Cylindrical Mirror Analyser from the same company was used. Sputter experiments were conducted with argon ions of 0.5 µA sputter current. As argon gun a PHI 04-303 Ion Gun, also from Perkin Elmer Corp., Physical Electronics Division, was utilised. Between every AES measurement the surface was sputtered for 6 s.

After electrochemical preparation of the sample in the glovebox, the crystal was removed from the cell and rinsed carefully with dry acetonitrile to rinse of residues of the electrolyte. The whole process, as well as the transfer into the AES measurement chamber, was conducted in nitrogen atmosphere with less than 0.5 ppm water and oxygen.

Author Contributions

F.M.S planned and carried out the experiments, analysed the data, and wrote the first version of the manuscript. M.-K.H. and J.M. contributed to the experimental measurements. M.U.C. and L.A.K. helped with the analysis of the results and discussion of the data. *T.J. conceived the experiments and supervised the project. All authors contributed to the discussion section and the finalisation of the text and figures of the manuscript.
